# Supplementary material for: Metacontrol of decision-making strategies in human aging
Source: eLife. 2019 Aug 9;8:e49154. doi: 10.7554/eLife.49154 (PMC6703898; doi:10.7554/eLife.49154)
Supplement: Supplementary file 1. [file elife-49154-supp1.docx]

**Supplementary File 1: Tables for model parameters, model comparison and statistical results**

Bolenz, Kool, Reiter, & Eppinger: Metacontrol of decision-making strategies in human aging

| Table S1 | | | | |
| --- | --- | --- | --- | --- |
| *Parameter estimates in the exhaustive reinforcement learning model (mean (SD))* | | | | |
| Parameter | low stakes  stable | high stakes  stable | low stakes  variable | high stakes  variable |
| inverse softmax temperature |  |  |  |  |
| *younger adults* | 0.46 (0.29) | 0.67 (0.21) | 0.46 (0.27) | 0.60 (0.24) |
| *older adults* | 0.58 (0.37) | 0.64 (0.39) | 0.50 (0.30) | 0.52 (0.25) |
| reward learning rate |  |  |  |  |
| *younger adults* | 0.78 (0.17) | 0.77 (0.19) | 0.69 (0.20) | 0.68 (0.19) |
| *older adults* | 0.48 (0.35) | 0.49 (0.34) | 0.44 (0.30) | 0.46 (0.30) |
| eligibility trace decay |  |  |  |  |
| *younger adults* | 0.57 (0.13) | 0.56 (0.13) | 0.54 (0.14) | 0.57 (0.15) |
| *older adults* | 0.52 (0.13) | 0.50 (0.16) | 0.53 (0.14) | 0.51 (0.18) |
| transition learning rate |  |  |  |  |
| *younger adults* | - | - | 0.55 (0.19) | 0.57 (0.19) |
| *older adults* | - | - | 0.39 (0.25) | 0.37 (0.21) |
| model-based weight |  |  |  |  |
| *younger adults* | 0.66 (0.21) | 0.75 (0.17) | 0.59 (0.18) | 0.66 (0.17) |
| *older adults* | 0.53 (0.25) | 0.52 (0.26) | 0.56 (0.21) | 0.57 (0.20) |
| choice stickiness |  |  |  |  |
| *younger adults* | 0.08 (0.52) | 0.26 (0.58) | 0.25 (0.54) | 0.37 (0.58) |
| *older adults* | 0.33 (0.75) | 0.47 (0.64) | 0.56 (0.79) | 0.64 (0.74) |
| response stickiness |  |  |  |  |
| *younger adults* | -0.18 (0.51) | -0.21 (0.38) | -0.22 (0.54) | -0.21 (0.51) |
| *older adults* | -0.28 (0.56) | -0.17 (0.52) | -0.17 (0.61) | -0.07 (0.63) |

| Table S2 | | | |
| --- | --- | --- | --- |
| *Parameter estimates in the standard reinforcement learning model* | | | |
| Parameter | Mean (SD)  younger adults | Mean (SD)  older adults | Effect-coded regression coefficient for age group:  Mean (credible interval) |
| inverse softmax temperature | 0.49 (0.17) | 0.54 (0.57) | -0.04 ([-0.20, 0.11]) |
| reward learning rate | 0.76 (0.28) | 0.55 (0.36) | 0.21 ([0.10, 0.32)] |
| eligibility trace decay | 0.55 (0.22) | 0.52 (0.22) | 0.03 ([-0.05, 0.11]) |
| transition learning rate | 0.58 (0.24) | 0.35 (0.23) | 0.22 ([0.14, 0.31]) |
| model-based weight (low stakes, stable transitions) | 0.62 (0.25) | 0.54 (0.24) | 0.08 ([-0.01, 0.17)] |
| model-based weight (high stakes, stable transitions) | 0.77 (0.15) | 0.54 (0.25) | 0.22 ([0.15, 0.30]) |
| model-based weight (low stakes, variable transitions) | 0.57 (0.19) | 0.56 (0.19) | 0.02 ([-0.06, 0.08]) |
| model-based weight (high stakes, variable transitions) | 0.64 (0.17) | 0.52 (0.18) | 0.12 ([0.05, 0.18]) |
| choice stickiness | 0.39 (0.63) | 0.83 (0.94) | -0.44 ([-0.73, -0.14]) |
| response stickiness | -0.27 (0.42) | -0.23 (0.59) | -0.05 ([-0.23, 0.14]) |

| Table S3 | | |
| --- | --- | --- |
| *Analysis of model-based weights (model with perfect transition learning, complete sample)* | | |
| Predictor (effect-coded) | $\bar{\beta}$ | 95% CI |
| intercept | 0.56 | [0.53, 0.58] |
| age group | 0.14 | [0.09, 0.19] |
| stakes | 0.06 | [0.02, 0.09] |
| transition stability | 0.12 | [0.09, 0.15] |
| age group * stakes | 0.12 | [0.06, 0.18] |
| age group * transition stability | 0.03 | [-0.03, 0.09] |
| stakes * transition stability | 0.04 | [-0.03, 0.10] |
| age group * stakes * transition stability | 0.06 | [-0.07, 0.18] |

| Table S4 | | | | | | | |
| --- | --- | --- | --- | --- | --- | --- | --- |
| *Model comparison results* | | | | | | | |
| Free model parameters | | | | | Akaike Information Criterion | | |
| *λ* | *η* | *η_CF_* | *π* | *ρ* | all | younger adults | older adults |
|  |  |  |  |  | 46977.0 | 21951.1 | 25025.9 |
| x |  |  |  |  | 46705.5 | 21807.2 | 24898.3 |
|  | x |  |  |  | 46653.2 | 21886.6 | 24766.6 |
| x | x |  |  |  | 46414.6 | 21762.6 | 24651.9 |
|  |  | x |  |  | 46993.1 | 21953.9 | 25039.2 |
| x |  | x |  |  | 46792.9 | 21845.2 | 24947.8 |
|  | x | x |  |  | 46910.5 | 22003.9 | 24906.5 |
| x | x | x |  |  | 46708.9 | 21891.6 | 24817.3 |
|  |  |  | x |  | 45964.6 | 21765.8 | 24198.8 |
| x |  |  | x |  | 45787.9 | 21658.9 | 24129.0 |
|  | x |  | x |  | 45728.8 | 21740.8 | 23987.9 |
| x | x |  | x |  | 45586.5 | 21646.1 | 23940.5 |
|  |  | x | x |  | 46039.9 | 21801.7 | 24238.2 |
| x |  | x | x |  | 45913.8 | 21719.7 | 24194.1 |
|  | x | x | x |  | 46021.4 | 21871.0 | 24150.5 |
| x | x | x | x |  | 45890.9 | 21786.8 | 24104.2 |
|  |  |  |  | x | 46745.7 | 21889.4 | 24856.3 |
| x |  |  |  | x | 46480.3 | 21751.4 | 24728.9 |
|  | x |  |  | x | 46428.1 | 21829.3 | 24598.8 |
| x | x |  |  | x | 46199.6 | 21709.2 | 24490.3 |
|  |  | x |  | x | 46769.3 | 21895.2 | 24874.2 |
| x |  | x |  | x | 46573.7 | 21792.0 | 24781.6 |
|  | x | x |  | x | 46691.5 | 21946.4 | 24745.1 |
| x | x | x |  | x | 46491.8 | 21838.4 | 24653.5 |
|  |  |  | x | x | 45746.2 | 21710.1 | 24036.1 |
| x |  |  | x | x | 45573.8 | 21607.2 | 23966.6 |
|  | x |  | x | x | 45514.4 | 21686.0 | 23828.3 |
| **x** | **x** |  | **x** | **x** | **45379.6** | **21597.4** | **23782.2** |
|  |  | x | x | x | 45825.9 | 21747.0 | 24078.9 |
| x |  | x | x | x | 45705.1 | 21670.4 | 24034.6 |
|  | x | x | x | x | 45809.7 | 21815.8 | 23993.8 |
| x | x | x | x | x | 45682.6 | 21738.5 | 23944.1 |

| Table S5 | | |
| --- | --- | --- |
| *Analysis of baseline-corrected reward (complete sample)* | | |
| Predictor (effect-coded) | $\bar{\beta}$ | 95% CI |
| intercept | 0.40 | [0.36, 0.44] |
| age group | 0.25 | [0.17, 0.33] |

| Table S6 | | |
| --- | --- | --- |
| *Analysis of model-based weights (standard model, complete sample)* | | |
| Predictor (effect-coded) | $\bar{\beta}$ | 95% CI |
| intercept | 0.59 | [0.57, 0.62] |
| age group | 0.11 | [0.05, 0.16] |
| stakes | 0.05 | [0.01, 0.08] |
| transition stability | 0.04 | [0.01, 0.07] |
| age group * stakes | 0.12 | [0.06, 0.18] |
| age group * transition stability | 0.09 | [0.03, 0.15] |
| stakes * transition stability | 0.06 | [-0.00, 0.12] |
| age group * stakes * transition stability | 0.04 | [-0.07, 0.16] |

| Table S7 | | |
| --- | --- | --- |
| *Analysis of model-based weights (exhaustive model, complete sample)* | | |
| Predictor (effect-coded) | $\bar{\beta}$ | 95% CI |
| intercept | 0.60 | [0.58, 0.63] |
| age group | 0.12 | [0.07, 0.17] |
| stakes | 0.04 | [0.01, 0.07] |
| transition stability | 0.02 | [-0.02, 0.05] |
| age group * stakes | 0.07 | [0.01, 0.14] |
| age group * transition stability | 0.12 | [0.06, 0.19] |
| stakes * transition stability | 0.00 | [-0.06, 0.06] |
| age group * stakes * transition stability | 0.05 | [-0.07, 0.17] |

| Table S8 | | |
| --- | --- | --- |
| *Analysis of transition learning rates (standard model, complete sample)* | | |
| Predictor (effect-coded) | $\bar{\beta}$ | 95% CI |
| intercept | 0.46 | [0.42, 0.50] |
| age group | 0.22 | [0.14, 0.31] |

| Table S9 | | |
| --- | --- | --- |
| *Analysis of model-based weights (standard model, performance-matched sample)* | | |
| Predictor (effect-coded) | $\bar{\beta}$ | 95% CI |
| intercept | 0.59 | [0.55, 0.63] |
| age group | 0.02 | [-0.06, 0.10] |
| stakes | 0.07 | [0.01, 0.12] |
| transition stability | 0.03 | [-0.02, 0.08] |
| age group * stakes | 0.17 | [0.07, 0.28] |
| age group * transition stability | 0.07 | [-0.04, 0.17] |
| stakes * transition stability | 0.02 | [-0.08, 0.13] |
| age group * stakes * transition stability | 0.15 | [-0.07, 0.36] |

| Table S10 | | |
| --- | --- | --- |
| *Analysis of model-based weights when controlling for baseline-corrected reward (standard model, complete sample)* | | |
| Predictor (effect-coded) | $\bar{\beta}$ | 95% CI |
| intercept | 0.44 | [0.40, 0.48] |
| age group | 0.01 | [-0.04, 0.06] |
| stakes | 0.13 | [0.07, 0.19] |
| transition stability | -0.08 | [-0.14, -0.02] |
| reward | 0.39 | [0.30, 0.49] |
| age group * stakes | 0.18 | [0.11, 0.25] |
| age group * transition stability | 0.01 | [-0.06, 0.07] |
| stakes * transition stability | 0.12 | [0.00, 0.23] |
| points * stakes | -0.21 | [-0.34, -0.08] |
| points * transition stability | 0.31 | [0.18, 0.44] |
| age group * stakes * transition stability | -0.08 | [-0.05, 0.21] |
| points * stakes * transition stability | -0.15 | [-0.41, 0.11] |

| Table S11 | | |
| --- | --- | --- |
| *Analysis of log second-stage reaction times in variable-transitions blocks (complete sample)* | | |
| Predictor (dummy-coded) | $\bar{\beta}$ | 95% CI |
| intercept | -0.76 | [-0.81, -0.72] |
| age group (1 = older adults) | 0.32 | [0.26, 0.39] |
| revaluation trial (1 = yes) | 0.15 | [0.11, 0.18] |
| age group * revaluation trial | -0.09 | [-0.14, -0.05] |

| Table S12 | | |
| --- | --- | --- |
| *Analysis of log second-stage reaction times in variable-transitions blocks when controlling for reward expectation (standard model, complete sample)* | | |
| Predictor (dummy-coded) | $\bar{\beta}$ | 95% CI |
| intercept | -0.76 | [-0.81, -0.72] |
| age group (1 = older adults) | 0.33 | [0.26, 0.40] |
| revaluation trial (1 = yes) | 0.15 | [0.11, 0.18] |
| reward expectation | -0.01 | [-0.02, -0.00] |
| age group * revaluation trial | -0.09 | [-0.14, -0.05] |
| age group * reward expectation | 0.01 | [-0.01, 0.02] |

| Table S13 | | |
| --- | --- | --- |
| *Analysis of log second-stage reaction times in variable-transitions blocks as a function of model-based control in low-stakes trials (standard model, complete sample)* | | |
| Predictor (dummy-coded) | $\bar{\beta}$ | 95% CI |
| intercept | -0.63 | [-0.76, -0.51] |
| revaluation trial (1 = yes) | 0.01 | [-0.07, 0.08] |
| model-based weight (low-stakes, variable-transitions) | 0.04 | [-0.18, 0.27] |
| revaluation trial * model-based weight (low-stakes, variable-transitions) | 0.17 | [0.04, 0.30] |

| Table S14 | | |
| --- | --- | --- |
| *Analysis of log second-stage reaction times in variable-transitions blocks as a function of model-based control in low-stakes trials (standard model, younger adults)* | | |
| Predictor (dummy-coded) | $\bar{\beta}$ | 95% CI |
| intercept | -0.85 | [-1.00, -0.70] |
| revaluation trial (1 = yes) | 0.07 | [-0.05, 0.18] |
| model-based weight (low-stakes, variable-transitions) | 0.15 | [-0.10, 0.39] |
| revaluation trial * model-based weight (low-stakes, variable-transitions) | 0.14 | [-0.05, 0.33] |

| Table S15 | | |
| --- | --- | --- |
| *Analysis of log second-stage reaction times in variable-transitions blocks as a function of model-based control in low-stakes trials (standard model, older adults)* | | |
| Predictor (dummy-coded) | $\bar{\beta}$ | 95% CI |
| intercept | -0.44 | [-0.60, -0.27] |
| revaluation trial (1 = yes) | -0.04 | [-0.13, 0.06] |
| model-based weight (low-stakes, variable-transitions) | 0.00 | [-0.27, 0.28] |
| revaluation trial * model-based weight (low-stakes, variable-transitions) | 0.17 | [0.01, 0.33] |

| Table S16 | | |
| --- | --- | --- |
| *Analysis of log second-stage reaction times in variable-transitions blocks as a function of model-based control in high-stakes trials (standard model, complete sample)* | | |
| Predictor (dummy-coded) | $\bar{\beta}$ | 95% CI |
| intercept | -0.37 | [-0.51, -0.24] |
| revaluation trial (1 = yes) | -0.02 | [-0.10, 0.06] |
| model-based weight (high-stakes, variable-transitions) | -0.40 | [-0.63, -0.16] |
| revaluation trial * model-based weight (high-stakes, variable-transitions) | 0.20 | [0.07, 0.34] |

| Table S17 | | |
| --- | --- | --- |
| *Analysis of log second-stage reaction times in variable-transitions blocks as a function of model-based control in high-stakes trials (standard model, younger adults)* | | |
| Predictor (dummy-coded) | $\bar{\beta}$ | 95% CI |
| intercept | -0.70 | [-0.87, -0.53] |
| revaluation trial (1 = yes) | 0.08 | [-0.06, 0.22] |
| model-based weight (high-stakes, variable-transitions) | -0.09 | [-0.36, 0.16] |
| revaluation trial * model-based weight (high-stakes, variable-transitions) | 0.11 | [-0.10, 0.32] |

| Table S18 | | |
| --- | --- | --- |
| *Analysis of log second-stage reaction times in variable-transitions blocks as a function of model-based control in high-stakes trials (standard model, older adults)* | | |
| Predictor (dummy-coded) | $\bar{\beta}$ | 95% CI |
| intercept | -0.35 | [-0.51, -0.19] |
| revaluation trial (1 = yes) | -0.03 | [-0.13, 0.07] |
| model-based weight (high-stakes, variable-transitions) | -0.17 | [-0.47, 0.14] |
| revaluation trial * model-based weight (high-stakes, variable-transitions) | 0.16 | [-0.02, 0.34] |

| Table S19 | | |
| --- | --- | --- |
| *Analysis of inverse softmax temperature (exhaustive model, older adults)* | | |
| Predictor (effect-coded) | $\bar{\beta}$ | 95% CI |
| intercept | 0.56 | [0.49, 0.63] |
| stakes | 0.04 | [-0.02, 0.10] |
| transition stability | 0.10 | [0.04, 0.16] |
| stakes * transition stability | 0.04 | [-0.08, 0.17] |

| Table S20 | | |
| --- | --- | --- |
| *Analysis of choice stickiness (exhaustive model, older adults)* | | |
| Predictor (effect-coded) | $\bar{\beta}$ | 95% CI |
| intercept | 0.50 | [0.35, 0.65] |
| stakes | 0.11 | [-0.02, 0.24] |
| transition stability | -0.21 | [-0.34, -0.07] |
| stakes * transition stability | 0.07 | [-0.20, 0.33] |

| Table S21 | | |
| --- | --- | --- |
| *Analysis of log reaction times in the task-switching task (complete sample)* | | |
| Predictor | $\bar{\beta}$ | 95% CI |
| intercept | -0.74 | [-0.77, -0.72] |
| age group (1 = older adults) | 0.36 | [0.32, 0.39] |
| reward cue duration | 0.00 | [-0.00, 0.00] |
| trial type (1 = switch) | 0.02 | [0.02, 0.03] |
| task (1 = size) | 0.01 | [0.00, 0.03] |
| previous trial type (1 = switch) | 0.00 | [-0.01, 0.01] |
| trial number | -0.02 | [-0.03, -0.01] |
| previous error (1 = yes) | 0.05 | [0.04, 0.06] |
| same response (1 = yes) | -0.01 | [-0.02, -0.00] |
| points at stake | 0.00 | [-0.00, 0.01] |
| points at stake * age group | -0.01 | [-0.01, 0.00] |
